# Quantitative Proteomics Reveals Changes Induced by TIMP-3 on Cell Membrane Composition and Novel Metalloprotease Substrates

**DOI:** 10.3390/ijms22052392

**Published:** 2021-02-27

**Authors:** Anna Paola Carreca, Veronica Maria Pravatà, Danilo D’Apolito, Simone Bonelli, Matteo Calligaris, Elisa Monaca, Stephan A. Müller, Stefan F. Lichtenthaler, Simone Dario Scilabra

**Affiliations:** 1 Proteomics Group of Fondazione Ri.MED, Department of Research IRCCS ISMETT, via Ernesto Tricomi 5, 90145 Palermo, Italy; apcarreca@fondazionerimed.com (A.P.C.); sbonelli@fondazionerimed.com (S.B.); mcalligaris@Fondazionerimed.com (M.C.); 2 Division of Gene Regulation and Expression, School of Life Sciences, University of Dundee, Dundee DD1 5EH, UK; m.v.pravata@dundee.ac.uk; 3 German Center for Neurodegenerative Diseases (DZNE), Feodor-Lynen Strasse 17, 81377 Munich, Germany; Stephan.Mueller@dzne.de (S.A.M.); Stefan.Lichtenthaler@dzne.de (S.F.L.); 4 Unità di Medicina di Laboratorio e Biotecnologie Avanzate, IRCCS-ISMETT (Istituto Mediterraneo per i Trapianti e Terapie ad Alta Specializzazione), Via E. Tricomi 5, 90127 Palermo, Italy; ddapolito@fondazionerimed.com; 5 Unità Prodotti Cellulari (GMP), Fondazione Ri.MED c/o IRCCS-ISMETT, Via E. Tricomi 5, 90127 Palermo, Italy; 6 Department of Pharmacy, University of Pisa, Via Bonanno 6, 56126 Pisa, Italy; 7 Fondazione Ri.MED, 90133 Palermo, Italy; emonaca@fondazionerimed.com; 8 Neuroproteomics, Klinikum rechts der Isar, Technische Universität München, 81675 Munich, Germany; 9 Munich Cluster for Systems Neurology (SyNergy), 81377 Munich, Germany

**Keywords:** tissue inhibitor of metalloproteases 3 (TIMP-3), metalloproteinases, ectodomain shedding, proteomics

## Abstract

Ectodomain shedding is a key mechanism of several biological processes, including cell-communication. Disintegrin and metalloproteinases (ADAMs), together with the membrane-type matrix metalloproteinases, play a pivotal role in shedding transmembrane proteins. Aberrant shedding is associated to several pathological conditions, including arthritis. Tissue inhibitor of metalloproteases 3 (TIMP-3), an endogenous inhibitor of ADAMs and matrix metalloproteases (MMPs), has been proven to be beneficial in such diseases. Thus, strategies to increase TIMP-3 bioavailability in the tissue have been sought for development of therapeutics. Nevertheless, high levels of TIMP-3 may lead to mechanism-based side-effects, as its overall effects on cell behavior are still unknown. In this study, we used a high-resolution mass-spectrometry-based workflow to analyze alterations induced by sustained expression of TIMP-3 in the cell surfaceome. In agreement with its multifunctional properties, TIMP-3 induced changes on the protein composition of the cell surface. We found that TIMP-3 had differential effects on metalloproteinase substrates, with several that accumulated in TIMP-3-overexpressing cells. In addition, our study identified potentially novel ADAM substrates, including ADAM15, whose levels at the cell surface are regulated by the inhibitor. In conclusion, our study reveals that high levels of TIMP-3 induce modifications in the cell surfaceome and identifies molecular pathways that can be deregulated via TIMP-3-based therapies.

## 1. Introduction

The proteolytic cleavage of transmembrane proteins and the consequent release of their ectodomains in the extracellular milieu, known as ectodomain shedding, is a key mechanism in several biological processes, including cell communication, adhesion, and transport [1]. About 2% of transmembrane proteins undergo ectodomain shedding, with the disintegrin and metalloproteinases (ADAMs) and the membrane-type members of the matrix metalloprotease family (MT-MMPs) playing a major role in this process [2,3]. The activity of ADAMs and MT-MMPs, as well as subsequent ectodomain shedding, have to be finely regulated. This occurs at several levels, including inhibition by the tissue inhibitors of metalloproteases (TIMPs) [4]. Differently from the other three mammalian TIMPs, which have a very restricted inhibitory profile against ADAMs, TIMP-3 can inhibit several members of this family, including ADAM10, 12, 17, 28, and 33 [4]. In addition, TIMP-3 is able to inhibit the matrix metalloproteinases (MMPs), including the membrane-type MMPs that represent another major class of proteinases involved in ectodomain shedding. Uniquely among TIMPs, TIMP-3 is also able to inhibit members of the related family of disintegrin and metalloproteinases with thrombospondin motifs (ADAMTSs) [4].

Aberrant cell-surface proteolysis has detrimental effects on cell behavior and is associated with the development of several pathological conditions, including inflammatory diseases and cancer [1]. For its ability to modulate ectodomain shedding, in addition to control ECM turnover, TIMP-3 has been proven protective in a number of diseases. Ablation of TIMP-3 in mouse, and subsequent loss of ADAM17 inhibition, leads to arthritis, chronic hepatic inflammation, insulin resistance, and liver/heart failure due to excessive release of tumor necrosis factor (TNF) [5,6,7]. Furthermore, aberrant ADAM10 activity in TIMP-3 deficient mice promoted liver failure in a model of hepatic ischemia/reperfusion injury [8]. TIMP-3 is often silenced in malignancies and its deficiency in mouse enhances tumor progression [9]. Conversely, by targeting ADAM17, there is an overexpression of TIMP-3 dampened TNF-release that ameliorated the development of arthritis in a murine model of the disease [10]. Similarly, TIMP-3 overexpression in mouse macrophages reduced adipose inflammation, insulin resistance, nonalcoholic fatty liver disease, and atherosclerotic plaques in an atherosclerosis mouse model [11,12]. Transgenic overexpression of TIMP-3, as well as treatment with the purified protein, reduced cancer progression in vivo [13]. As a consequence of its beneficial effects, strategies to increase TIMP-3 levels in the tissue have been widely sought as a potential therapeutic treatment for diseases characterized by enhanced proteolysis. Nevertheless, overall effects of increased TIMP-3 on cell behavior are unknown, and it is difficult to predict whether TIMP-3-based therapies may lead to mechanism-based side effects due to high levels of the inhibitor. 

Unbiased proteomics allows one to perform a comprehensive analysis of molecular pathways that can be affected by high levels of TIMP-3 and predict potential side effects that may be induced by TIMP-3-based therapies. In this study, we generated a TIMP-3 stably transfected cell line, in which sustained expression of the inhibitor was maintained by using hygromycin B as a selection reagent. As such, we developed a high-resolution mass spectrometry-based workflow to evaluate changes induced by the overexpression of TIMP-3 in the cell surface proteome. Our analysis revealed that TIMP-3 induced significant changes in the cell surfaceome, which is in line with its ability to inhibit several members of ADAMs and other classes of metalloproteinases. Several, but not all, known metalloproteinase substrates accumulated on the cell surface of TIMP-3 overexpressing cells. In addition, we found a number of type-1 transmembrane proteins, including ADAM15, whose levels were increased in response to TIMP-3, that can be novel ADAM substrates. Furthermore, we discovered that hygromycin B as a selection reagent can be used for stably transfected cells that have unexpected effects on cells, including altering stability of specific proteins, as we demonstrated on the ephrin type-A receptor 4 (EphA4). 

In conclusion, this study provides a comprehensive analysis of modifications occurring in the cell surfaceome as a consequence of increased expression of TIMP-3, identifies proteins that accumulate at the cell surface, including potentially novel ADAM substrates, and molecular pathways that may be affected by increased levels of TIMP-3. Furthermore, it can serve as a valuable tool to predict mechanism-based side effects that may arise from TIMP-3-based therapies and to analyze functions of proteases and inhibitors in a comprehensive cell context-dependent manner. 

## 2. Results

### 2.1. Surfaceome Analysis of TIMP-3 Overexpressing Cells

In order to identify alterations induced by TIMP-3 in the cell surface proteome, we stably transfected HEK 293 cells to generate a TIMP-3 overexpressing cell line (TIMP-3/HEK) and developed a high-resolution mass spectrometry-based workflow to perform an unbiased quantitative analysis of cell surface protein levels in these cells. HEK 293 cells express high levels of ADAMs and therefore they have high shedding potential. For this reason, HEK 293 cells are commonly utilized as a preliminary model to investigate shedding of transmembrane proteins with strong implications in disease, including Alzheimer’s-related amyloid precursor protein (APP) [14]. Cell membrane proteins of TIMP-3/HEK or control HEK 293 cells were labelled using Sulfo-NHS-biotin and isolated through streptavidin pull-down. 

**Figure 1:**
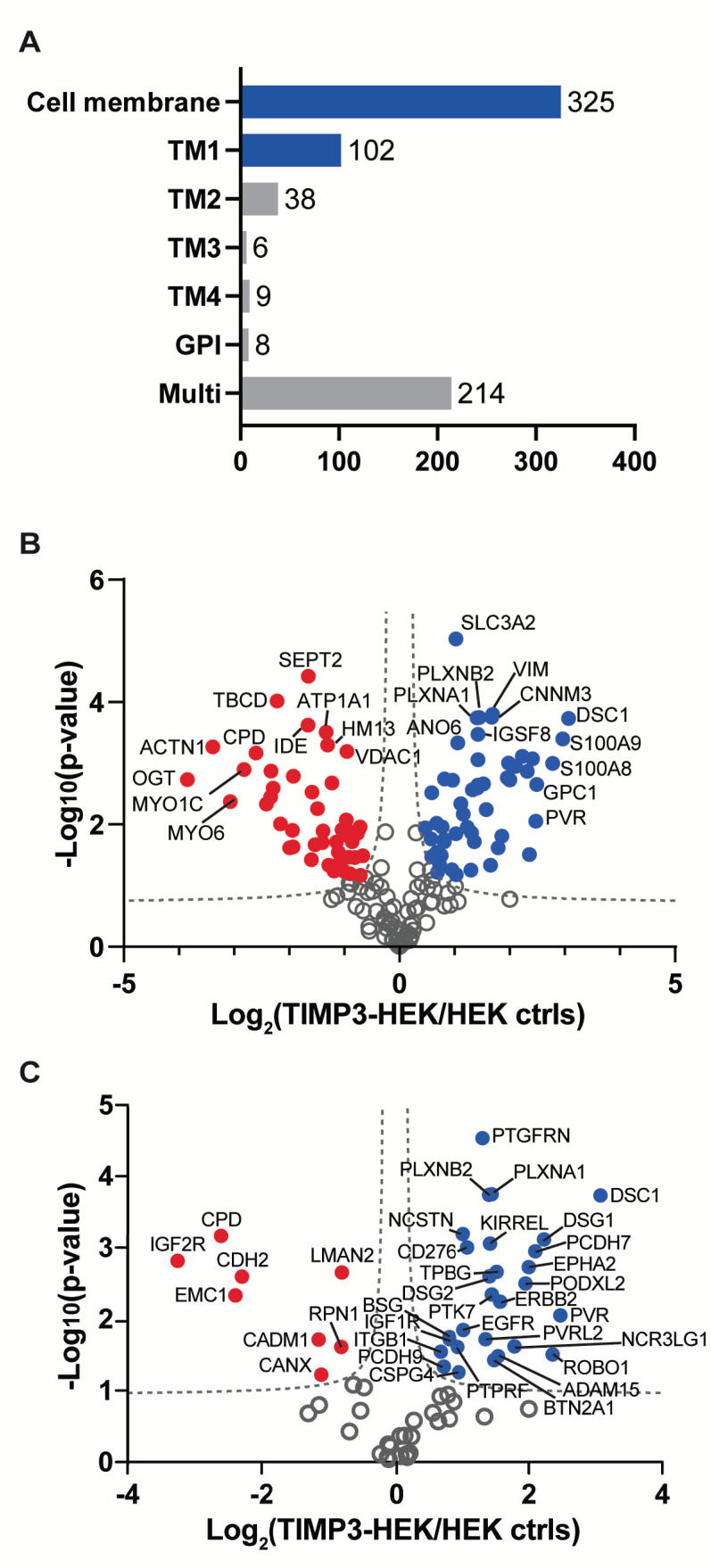
Analysis of cell membrane composition of TIMP-3 overexpressing cells. (**A**) Number and topology of membrane proteins detected in the surfaceome of TIMP-3/HEK cells. (**B**) Volcano plot showing the -log10 of *p*-values versus the log2 of protein ratio between TIMP-3 overexpressing HEK 293 cells (TIMP-3-HEK) and control HEK 293 cells (HEK ctrls) of 325 membrane proteins (*n* = 3). Proteins significantly regulated are displayed as the filled dots above the false discovery curves (grey dashed hyperbolic curves; computed by Perseus software with FDR *p* = 0.05; s_0_ = 0.1). Red dots correspond to less abundant proteins, blue dots to more abundant proteins in TIMP-3/HEK cells. (**C**) Volcano plot showing the 102 type-1 proteins detected in the cell membrane of TIMP-3/HEK cells.

These proteins were analyzed via LC-MS/MS and levels assessed through label-free quantification. The analysis detected 3052 proteins, of which 325 cell membrane proteins were comprised of both integral and peripheral membrane proteins, based on UniProt annotation (Figure 1A, Supplementary Table S1). In addition to cell membrane proteins, the mass-spectrometry analysis detected intracellular proteins. This is in line with previous reports, as the cytoskeletal network that forms strong physical links between the plasma membrane and intracellular components is unspecific when binding to the streptavidin resin. Further, biotin acts as a cofactor of carboxylases and, to a certain extent, cell penetration of the reagent acts in spite of its negatively charged sulpho group [15,16]. Nevertheless, enrichment of cell membrane proteins reduces sample complexity and allows mass-spectrometry detection of low abundant membrane proteins that would not be detected in a whole cellular proteome analysis, as their signal would be covered by that of high abundant intracellular proteins [15,17]. Proteins above the false discovery rate curves (shown as dashed grey hyperbolic curves in Figure 1B and 1C; FDR *p* = 0.05; s_0_ = 0.1) were considered as differentially abundant proteins. Moreover, 30.15% of the cell membrane proteins were significantly regulated in TIMP-3/HEK cells, further indicating the multifunctional properties of TIMP-3. Among them, 45 were less abundant in TIMP-3/HEK and, conversely, 53 proteins were increased in TIMP-3/HEK, such as glypican (GPC1), protein S100A8, and S100A9 that together formed the calprotectin complex (Figure 1B, Supplementary Table S1). In addition, the analysis detected 102 transmembrane type-1 proteins, 38 type-2, 214 multi-pass transmembrane proteins, and 23 other proteins with different topologies (Figure 1A, Supplementary Table S1). ADAMs and MT-MMPs, of which TIMP-3 is an endogenous inhibitor, mainly cleave type 1 transmembrane proteins, thereby promoting release of their ectodomains [1]. For this reason, we focused our analysis on the 102 transmembrane type-1 proteins. In total, 28 of these proteins significantly accumulated on the cell surface of TIMP-3/HEK (Figure 1C, Table 1), which is in agreement with reduced metalloproteinase-mediated shedding in the presence of high levels of TIMP-3. As expected, several of these proteins were known to undergo metalloproteinase-dependent shedding, including inactive tyrosine-protein kinase 7 (PTK7), podocalyxin-like protein 2 (PODXL2), and desmoglein 2 (DSG2) (Figure 1C, Table 1) [18,19]. Interestingly, not all ADAM substrates increased upon TIMP-3 overexpression. Indeed, a number of adhesion molecules (CADM1, MCAM, ALCAM, NCAM1, and CADM4), integrins (ITGA6, ITGA2, ITGAV, ITGA5, and ITGA1), ephrin proteins, and receptors (EPHA7, EPHB2, EFNB1, and EPHB4), as well as other proteins known to be ADAM substrates (SDC4, PROCR, etc.), did not accumulate on the cell surface of TIMP-3/HEK cells (Supplementary Table S1). Other than known ADAM substrates, 13 additional transmembrane type-1 proteins accumulated on the cell surface of TIMP-3/HEK, which could potentially be novel substrates of ADAMs or other metalloproteinases: DSC1, PVR, DSG1, PCDH7, ADAM15, TPBG, BTN2A1, KIRREL, PLXNA1, PVRL2, NCSTN, EGFR, and PTGFRN (Table 1). In total, 153 proteins were not statistically evaluated, as they were not identified in at least 2 biological replicates of TIMP-3/HEK and control cells (Supplementary Table S1). Among these, the cell membrane proteome analysis identified a number of ADAM substrates in the control HEK 293 cells that, rather than accumulating, were not present in TIMP-3/HEK. This group had two ADAM10 substrates, EphA4 and LRP-1 (Supplementary Table S1) [20,21].

In conclusion, our mass spectrometry-based analysis identified major changes induced by TIMP-3 in the cell surfaceome. As expected from increased metalloproteinase inhibition, several metalloproteinase substrates accumulated on the cell surface, as well as other proteins that could be novel ADAM substrates. Interestingly, another group of known ADAM substrates, including a number of adhesion molecules, integrins, and ephrin proteins, did not increase on the cell surface of TIMP-3/HEK.

### 2.2. Validation of the Surfaceome Analysis

**Figure 2:**
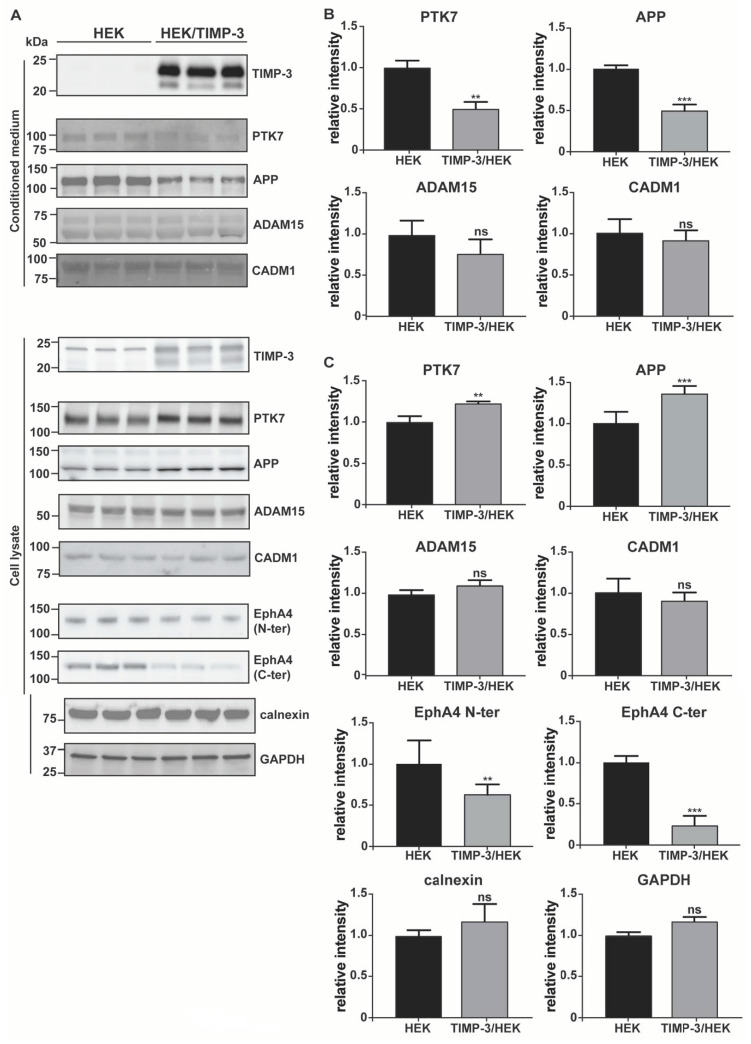
Analysis of protein changes by immunoblotting. (**A**) Immunoblots showing protein abundance of TIMP-3, PTK7, APP, ADAM15, and CADM1 in the conditioned media of TIMP-3/HEK and control HEK 293 cells, as well as levels of PTK7, APP, ADAM15, CADM1 and EphA4 in the cell lysates. Calnexin and GAPDH were used as loading controls. Densitometric quantifications of specific proteins in the conditioned media (**B**) and cell lysates (**C**) are displayed as mean values ± standard deviation (** *p* < 0.01, *** *p* < 0.005, Student’s t-test; from 3 to 6 separate experiments have been performed and analyzed).

**Figure 3:**
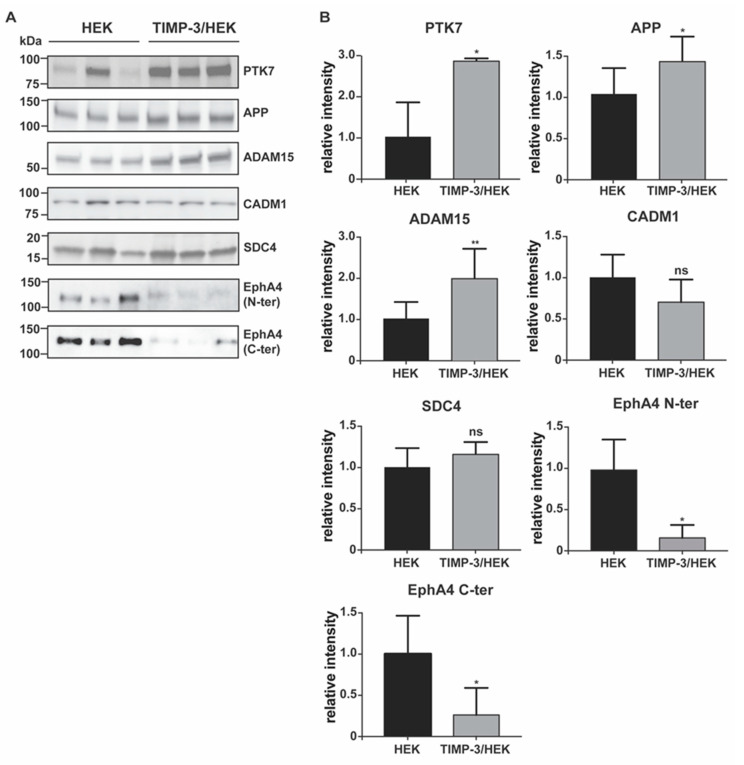
Validation of TIMP-3/HEK cell membrane proteome analysis by immunoblotting. Immunoblots (**A**) and their respective quantification (**B**) showing levels of PTK7, APP, ADAM15, CADM1, SDC4, and EphA4 in membrane proteins isolated from TIMP-3/HEK and control HEK 293 cells, (densitometric quantifications shown as mean values ± standard deviation; * *p* < 0.05, ** *p* < 0.01, Student’s t-test; from 3 to 6 separate experiments have been performed and analyzed).

**Figure 4:**
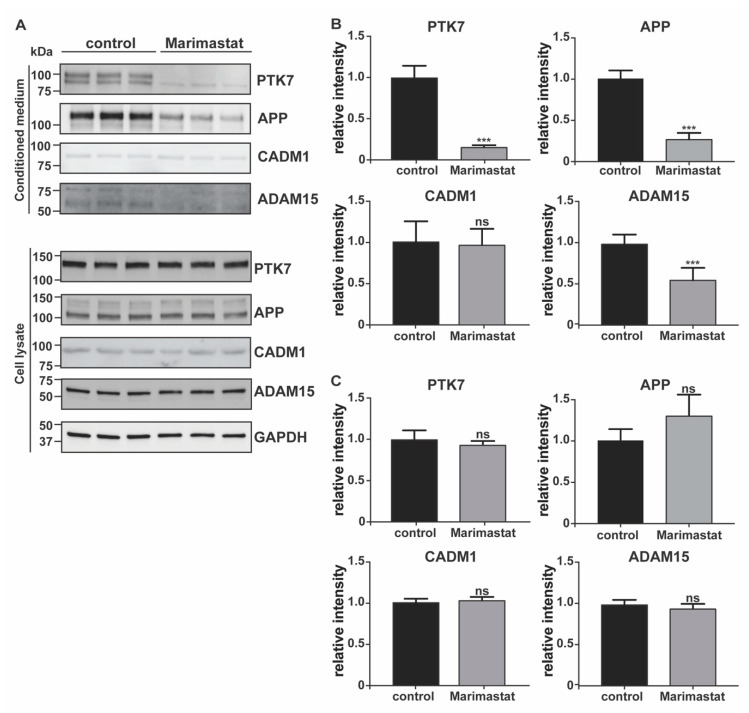
Validation of metalloprotease-dependent shedding of TIMP-3 regulated proteins by immunoblotting. Immunoblots (**A**) and their respective quantification (**B**, conditioned media; **C**, cell lysates) showing levels of PTK7, APP, CADM1, and ADAM15 in conditioned media or lysates of HEK 293 cells treated with or without marimastat (control). GAPDH was used as a loading control. Densitometric quantifications shown as mean values  ±  standard deviation; *** *p* < 0.005, Student’s t-test; (*n* = 6).

In order to validate results of the surfaceome analysis, we evaluated the changes of a number of TIMP-3 regulated transmembrane proteins using Western blotting as an orthogonal method. TIMP-3 is a secreted protein that inhibits metalloproteases when in the extracellular milieu. Indeed, the large majority of metalloproteases were synthesized in an inactive form comprising a pro-domain that prevents TIMP-3 binding before its removal. This occurred either in the late stages of the secretory pathway or after secretion in the extracellular milieu [32,33]. In this study, we aimed to identify consequences of TIMP-3 overexpression; thus, we first confirmed that TIMP-3 accumulated in the conditioned media of TIMP-3/HEK cells (Figure 2A; levels of TIMP-3 in the respective cell lysates are shown in Supplementary Materials). As a consequence of TIMP-3 overexpression and metalloprotease inhibition, levels of PTK7 decreased in the conditioned media and increased in the cell lysate of TIMP-3/HEK compared to the controls, similarly to those of APP, another well-characterized ADAM-substrate was used as a control (Figure 2) [14]. Proteomic analysis identified ADAM15 as a protein regulated by TIMP-3 and thus as a potential ADAM substrate. Nevertheless, its levels were altered neither in the cell lysate nor in the conditioned media of TIMP-3/HEK (Figure 2). For this reason, we isolated cell membrane proteins through biotinylation and streptavidin pulldown similarly to how as it was done within the proteomic workflow. In line with the proteomics results, levels of ADAM15 at the cell surface increased in TIMP-3/HEK, as well as those of the ADAM substrates PTK7 and APP (Figure 3). Treatment of HEK 293 cells with marimastat, a synthetic metalloproteinase inhibitor, clearly reduced shedding of ADAM15, in a similar manner as PTK7 and APP (Figure 4). This indicates that ADAM15 undergoes metalloprotease-dependent shedding and that TIMP-3 can stabilize it at the cell surface by an additional unknown mechanism.

Proteomics found that levels of the ADAM10 substrate CADM1 slightly decreased at the cell surface of TIMP-3/HEK cells rather than accumulating, as was expected by higher TIMP-3 expression and metalloprotease inhibition (Figure 1C, Supplementary Table S1) [34]. Immunoblotting showed that both TIMP-3 and marimastat did not prevent CADM1 shedding under these experimental conditions (Figure 2 and Figure 4), and its decrease on the cell membrane did not reach statistical significance (Figure 3). This confirmed the proteomic results that the ADAM substrate CADM1 does not accumulate at the cell surface upon TIMP-3 overexpression. In line with the surfaceome analysis, levels of SDC4, another protein that undergoes metalloproteinase-dependent, and TIMP-3-sensitive shedding, was not altered on the cell surface of TIMP-3/HEK (Supplementary Table S1, Figure 3), further indicating that not all metalloproteinase substrates effectively accumulated at the cell surface in response to increased levels of the inhibitor [35]. Finally, we analyzed via Western blotting levels of EphA4, an ADAM10 substrate that the mass-spectrometry-based analysis consistently quantified in HEK control cells, but it was below the limit of quantification in TIMP-3/HEK cells (Supplementary Table S1). Western blotting with two different antibodies, targeting the N-terminal or the C-terminal domain of EphA4, confirmed the mass-spectrometry data in that levels of the protein in both lysates (Figure 2A,C) and cell membranes (Figure 3) of TIMP-3 overexpressing cells markedly decreased compared to HEK control cells. 

### 2.3. Hygromycin B Promotes Decrease of EphA4 Levels

**Figure 5:**
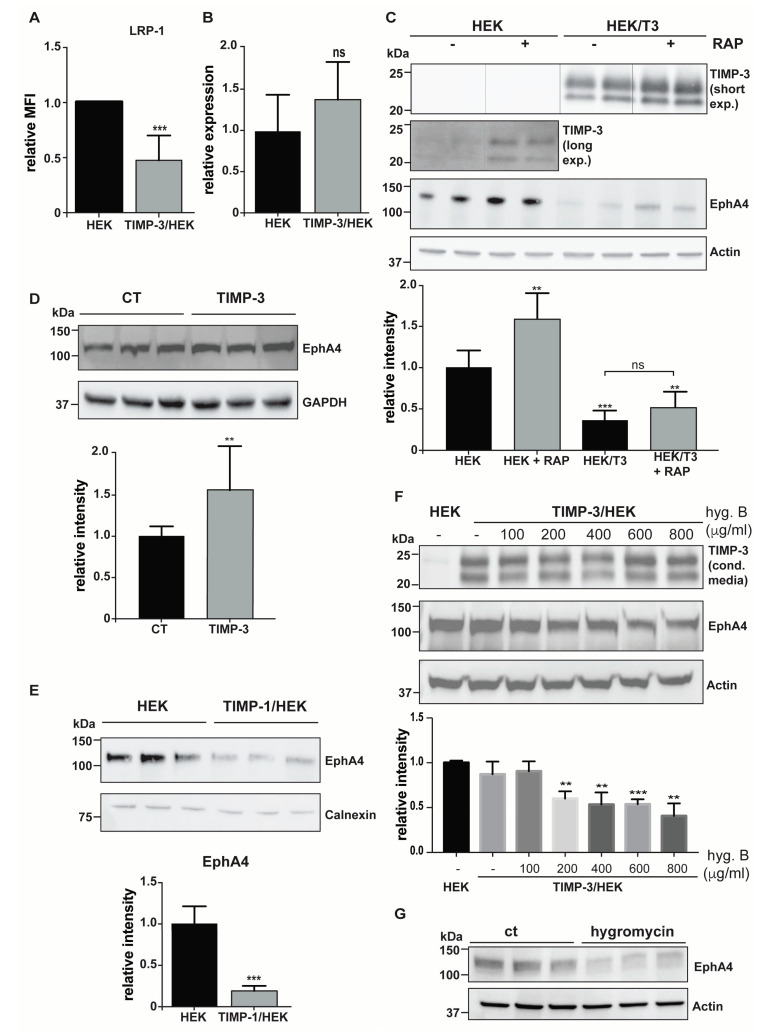
Treatment with hygromycin B of transgenic cells lowers levels of EphA4. (**A**) Levels of LRP-1 on TIMP-3/HEK or control cells was measured by flow cytometry and plotted as the average of mean fluorescent intensities (MFI) ± standard deviation (*n* = 3). (**B**) Expression levels of EphA4 mRNA in TIMP-3/HEK and control HEK 293 cells plotted as relative 2^−ΔΔCT^ (*n =* 4) (**C**) Immunoblots and their respective quantification (*n* = 4) showing levels of EphA4 in TIMP-3/HEK or control HEK 293 cell lysates, in the absence or presence of 500 nM RAP. Actin was used as a loading control. (**D**) Immunoblots and their respective quantification (*n* = 9) showed levels of EphA4 in lysates of HEK 293 cells supplemented with TIMP-3-containing or control conditioned media. GAPDH was used as a lading control. (**E**) Immunoblots and their respective quantification (*n* = 3) showed levels of EphA4 TIMP-1 overexpressing (TIMP-1/HEK) or control HEK 293 cells. Calnexin was used as a loading control. (**F**) Immunoblots and their respective quantification (*n* = 3) showed levels of TIMP-3 in the conditioned media, and EphA4 in the lysate of TIMP-3/HEK or control HEK 293 cells treated with different concentrations of hygromycin B (0–800 μg/mL). Actin was used as a loading control. (**G**) Immunoblots showed levels of EphA4 in the cell lysate of HEK 293 cells, transfected with pCEP4 and treated with or without 400 μg/mL hygromycin B. Actin was used a loading control. All densitometric quantifications shown as mean values ± standard deviation; (** *p* < 0.01, *** *p* < 0.005, Student’s t-test).

A number of proteins were only identified in control HEKs, but not in the surfaceome of TIMP-3 overexpressing cells (Supplementary Table S1). Among these proteins, LRP-1 and EphA4 are well characterized ADAM10 substrates, and therefore were expected to accumulate on the cell surface as a consequence of increased TIMP-3 expression [20,21]. LRP-1 decreased on the cell surface, which was validated via flow cytometry (Figure 5A), may potentially be provoked by sustained endocytosis of TIMP-3, one of the LRP-1 ligands with highest affinity for the receptor [36]. However, the molecular mechanism leading to a decrease of EphA4 levels remains elusive. Thus, we further investigated the molecular mechanism leading to this unpredicted phenotype of TIMP-3/HEK cells. EphA4 transcript levels were indistinguishable in TIMP-3/HEK or control cells, indicating that EphA4 was post-translationally regulated in TIMP-3/HEKs (Figure 5B). We previously found that TIMP-3 accelerated the endocytosis of LRP-1 ligands with a lower affinity for the endocytic receptor [36]. Given that another member of the ephrin-type A receptors, EphA2, associates with LRP-1, we hypothesized that LRP-1 could regulate levels of EphA4, with TIMP-3 being able to accelerate the process [37]. For this reason, we used RAP, an extensively characterized LRP-1 inhibitor, to test whether EphA4 could be endocytosed by LRP-1 through a process involving TIMP-3 [38]. RAP effectively blocked LRP-1-mediated endocytosis, thereby slowing down TIMP-3 turnover and promoting its accumulation in the conditioned media, both in TIMP-3/HEK and control cells (Figure 5C). Similarly, RAP increased levels of EphA4, suggesting it as a novel LRP-1 ligand. Nevertheless, RAP failed to rescue levels of EphA4 in TIMP-3/HEK, indicating that its endocytosis and subsequent degradation are not dependent on TIMP-3 (Figure 5C). We previously characterized EphA4 as an ADAM10 substrate, whose shedding was clearly inhibited when cells were treated with the ADAM10 inhibitor GI 254023X [21]. Similarly, addition of exogenous TIMP-3 to HEK 293 cells promoted accumulation of EphA4 compared to non-treated controls (Figure 5D). This was in contrast with EphA4 decrease in TIMP-3 overexpressing cells. We hypothesized that TIMP-3 could have an intracellular function, and to further address the discrepancy between TIMP-3 overexpression and its exogenous addition, we tested the effects on EphA4 of overexpression of TIMP-1, another TIMP that is also known to inhibit ADAM10 [39]. Similarly to TIMP-3, TIMP-1 overexpression led to a reduction of EphA4 levels compared to controls (Figure 5E), indicating that this mechanism is not specifically promoted by TIMP-3. TIMP-1 or TIMP-3 were inserted in the same vector carrying the hygromycin B resistance gene, and TIMP overexpressing cells were maintained in media supplemented with the same amount of the selection reagent before being used for experiments. Thus, we hypothesized that hygromycin B treatment could promote such a reduction in EphA4 levels. In order to confirm this hypothesis, we cultured TIMP-3 overexpressing HEK 293 cells with increasing concentrations of hygromycin B, and then levels of EphA4 were analyzed. No differences in viability between cells supplemented with Hygromycin B and non-treated controls were detected (Supplemental Materials). Maintaining TIMP-3/HEK cells in hygromycin B promoted decrease of EphA4 levels in a dose-dependent manner, with the selection reagent having an effect at concentrations as low as 200 μg/mL (Figure 5F). Furthermore, we transfected HEK 293 cells with an empty vector, carrying the hygromycin B resistance gene, but lacking the TIMP-3 sequence. When treated with hygromycin B, these cells displayed lower levels of EphA4 compared to control cells (Figure 5G). These results clearly indicated that hygromycin B can have unexpected effects on transgenic cells carrying the hygromycin B resistance gene. 

## 3. Discussion

Ectodomain shedding is a regulatory mechanism in which the ectodomain of transmembrane proteins is proteolytically released from the cell surface into the extracellular milieu. This mechanism plays a key role in a number of cellular processes, including cell-cell communication, immunity, adhesion, and cellular transport [1]. Several cytokines (TNF) and growth factors (EGF-like ligands, including TGFα, amphiregulin, and HB-EGF) are synthesized as transmembrane precursors that need to be shed from the cell surface in order to bind their specific receptor and trigger the signaling cascade [40]. In addition, surface levels and the function of other transmembrane proteins, including signaling receptors, endocytic receptors, and cell-adhesion molecules, can be regulated via ectodomain shedding [1]. The family of ADAMs is a major class of membrane-tethered metalloproteinases involved in the ectodomain shedding of membrane components [2]. In addition to ADAMs, MMPs play a significant role in this process. The membrane-type group of MMPs (MT-MMPs) possess a transmembrane domain that localize them in proximity of their ligands at the cell surface [3]. Other members of the MMP family, despite being secreted in the extracellular space, can bind to cell membrane components, including proteoglycans, and release transmembrane proteins [33]. 

Among the four mammalian TIMPs, TIMP-3 has the broader inhibitory profile, being able to inhibit several members of the ADAM family (including ADAM10, 12, 17, 28, and 33), MT-MMPs (including MT1-, MT3 and MT5-MMP), and soluble MMPs/ADAMTSs [4]. For this reason, TIMP-3 has been proven to be protective in a number of diseases characterized by enhanced proteolysis. Loss of TIMP-3 in mice leads to dysregulated TNF release and aberrant MMP and ADAMTS activity, thereby promoting increased cartilage breakdown in both models of inflammatory and surgically-induced arthritis, while articular injection of TIMP-3 ameliorated the pathology in a rat meniscal tear model of osteoarthritis [41,42]. TIMP-3 has been considered as a tumor suppressor in several human cancer types, as it inhibits cancer cell migration and invasion [13]. Ablation of TIMP-3 in mouse enhanced inflammation by dysregulating the activity of ADAM17 (a TIMP-3 target metalloproteinase) and the subsequent release of its substrate TNF, thus promoting insulin resistance and hepatosteatosis [5]. Conversely, by targeting the ADAM17/TNF axis, overexpression of TIMP-3 in mouse macrophages reduced adipose inflammation, insulin resistance, and nonalcoholic fatty liver disease, other than reducing atherosclerotic plaques in a mouse model of atherosclerosis [11,12,43]. Loss of TIMP-3 in mouse promoted aberrant shedding of E-cadherin by ADAM10, another TIMP-3 target metalloproteinase, and consequent disruption the E-cadherin/beta-catenin complex from the cell surface, thus provoking enhanced hepatocyte cell death and liver failure in a murine model of hepatic ischemia/reperfusion injury [8]. On these premises, it is clear that TIMP-3 has been considered as an excellent drug target in diseases characterized by enhanced proteolysis. There has been considerable interest in understanding factors regulating levels of the inhibitor in the tissue in order to develop therapeutics. Nevertheless, given that TIMP-3 is a multifunctional protein with several target metalloproteinases and binding partners, its therapeutic increase may lead to unwanted on-target side effects. 

In order to predict molecular pathways that can be deregulated in response to high levels of TIMP-3, we used high-resolution quantitative proteomics to analyze changes in the surfaceome of cells expressing high levels of the inhibitor. By using this method, we found that several, but not all metalloproteinase substrates, effectively accumulated at the cell surface of TIMP-3 overexpressing cells. Post-translational regulation of surface levels of a specific protein can be complex, as it does not depend only on shedding but also on its rate of secretion and endocytosis. In addition, ectodomain shedding itself can be regulated by multiple members of the metalloproteinase family, or involving members of other families of sheddases, such as BACE secretases and rhomboids [1,44]. Our study suggests that accumulation of some metalloproteinase substrates—and therefore specific molecular pathways—can be more responsive to TIMP-3 than others. Indeed, we found that a number of metalloproteinase substrates did not increase at the cell surface of HEK cells upon TIMP-3 overexpression. Among them, we characterized the ADAM substrate CADM1 [34]. Levels of CADM1 were slightly decreased on TIMP-3/HEK when measured by proteomics, although this decrease did not reach statistical significance when analyzed by immunoblotting (this small discrepancy between immunoblotting and proteomics can be due to the label-free quantification analysis, which is more accurate in recognizing little protein changes as it allows for the reduction of the variability between different biological replicates by normalizing their total protein amounts). Neither TIMP-3 nor a synthetic inhibitor of metalloproteases (marimastat) affected CADM1 shedding, suggesting that this process is not dependent on ADAMs, at least under these experimental conditions. Shedding of specific ADAM substrates can be different in different cells and in response to different stimuli. For example, acitretin stimulates ADAM10 to cleave APP in neurons, but not NrCAM, another well-known ADAM10 substrate [45]. The differential shedding of a specific ADAM substrate does not depend only on the protease activity but also on two groups of seven membrane spanning proteins, the tetraspanins and the inactive rhomboids, which have recently emerged as essential regulators of ADAM10 and ADAM17, respectively [46,47]. These protein regulators can integrate different cues, activate their cognate protease and address it towards specific groups of substrates [48]. Clearly, shedding regulation is highly multifactorial; thus, the proteomic approach that we used in this study can be a very powerful tool to investigate, in a comprehensive and cell context-dependent manner, the effects of sheddases and their inhibitors on cell behavior. 

In addition to known metalloproteinase substrates, we identified several other proteins that accumulated at the cell surface of TIMP-3 overexpressing cells. Within this group, there are potential novel metalloproteinase substrates and proteins that can be indirectly regulated by the inhibitor. Levels of PTGFRN, for instance, decrease in the secretome of TIMP-3 expressing cells, further indicating that these proteins can be novel metalloproteinase substrates, potentially ADAM10, as it resulted the most regulated proteinase by overexpression of the inhibitor [21]. Despite stabilizing ADAM15 on the cell surface, TIMP-3 overexpression did not reduce levels of shed ADAM15 in the conditioned medium. Nevertheless, the synthetic inhibitor of metalloproteases marimastat clearly inhibited its shedding, suggesting that ADAM15 can indeed undergo a metalloproteinase-dependent release, but that the mechanism by which TIMP-3 controls cell surface levels of ADAM15 may be different from its inhibition. We found it intriguing that levels of ADAM15, a sheddase itself, can be regulated by metalloproteinase-dependent shedding [49]. Although its substrate repertoire is quite limited, with only few proteins shown to be cleaved in vitro, ADAM15 is associated to development of age-related diseases, including osteoarthritis [50,51,52,53]. This indicates that the substrate spectrum, and therefore functions of this metalloproteinase, can be broader than what is currently known. It would be interesting to evaluate whether shedding of ADAM15 alters its substrate repertoire, in a similar manner as it does for ADAM10 and ADAM17, with the shed form of the enzyme being able to cleave different proteins from its membrane-tethered counterpart, thus eliciting its protective effects in osteoarthritis development [54].

Hygromycin B is an is an aminocyclitol antibiotic produced by *Streptomyces hygroscopicus* and developed in the 1950s as an anthelminthic agent in swine. In the early 1980s, gene sequence and enzymatic function of hygromycin B phosphotransferase (HPH) (the gene conferring resistance to the antibiotic) were characterized [55]. Since then, hygromycin B and its resistance gene *HPH* have been extensively used in cell biology to select transfected cells and ensure expression of transgenic genes. Hygromycin B is recommended for use as a selection agent at 100–800 μg/mL and its side effects are associated to the sustained treatment of transfected cells carrying the *HPH* transgene, which have not yet been reported in the literature. *HPH* expression in transgenic mice (referred to as hygR mice) causes resistance to toxic effects of hygromycin B in vivo and an 89-fold increase in the lethal dose compared to wild-type controls [56]. Nevertheless, at high doses, hygromycin B induced toxic effects in hygR transgenic mice, including acute inflammation, hepatocellular fatty change and necrosis, and acute tubular nephrosis, culminating in liver and kidney failure. In line with these results, our study displayed that sustained treatment with hygromycin B of stable transfected cells can lead to unwanted side effects. We found that EphA4, a well characterized ADAM10 substrate, unexpectedly was reduced in TIMP-3/HEK cells [21]. We demonstrated that such effects were not induced by TIMP-3, but by hygromycin B treatment. Considering that the hygromycin B *HPH* system is one of the most used in molecular biology to produce stable transfected cell lines, our results highlight the urge of a more comprehensive analysis of hygromycin B side effects. A proteomic analysis of transgenic cells expressing the *HPH* but no other transgene could be particularly suitable to evaluate such effects. 

In conclusion, TIMP-3 is beneficial in a number of diseases characterized by enhanced proteolysis. Thus, we developed a high-resolution mass spectrometry-based workflow to analyze alterations induced by TIMP-3 in the cell surfaceome and predict potential mechanism-based side-effects that can arise from TIMP-3-based therapies. We found that the inhibitor promoted major changes in the cell surfaceome, which is in line with its broad inhibitory profile and its various biological activities. We identified a number of proteins that accumulated at the cell surface in response to the increased expression of TIMP-3, suggesting specific molecular pathways that would be more responsive to high concentrations of the inhibitor and that could be potentially deregulated by TIMP-3-based therapies. 

## 4. Materials and Methods 

### 4.1. Generation of TIMP-3 and TIMP-1 Overexpressing Cells

Recombinant human C-terminally FLAG-tagged TIMP-3 or TIMP-1 was inserted in a pCEP4-based expression vector (Invitrogen, Loughborough, Leicestershire, UK) using the PCR method, as previously described [57]. Human embryonic kidney HEK293 cells were transfected with either TIMP-3 or TIMP-1 expression plasmid by lipofection with FuGENE6 (Roche Applied Science, Basel, Switzerland) and transfected cells selected by treatment with 800 μg/mL hygromycin B (Thermo Scientific, Waltham, MA, USA) over 3 weeks. Transfected cell lines were maintained under standard cell culture conditions in Dulbecco’s modified Eagle’s medium (DMEM) containing 10% (*v*/*v*) fetal calf serum, penicillin (100 units/mL), streptomycin (100 units/mL), and 800 μg/mL hygromycin B at 37 °C in 5% CO_2_, unless differently stated.

### 4.2. Cell Membrane Proteome Analysis of TIMP-3 Overexpressing Cells

TIMP-3/HEK were cultured in a complete medium supplemented with 800 μg/mL hygromycin B to ensure stable maintenance of the plasmid encoding TIMP-3 and its expression at high levels. TIMP-3/HEK or HEK 293 cells were seeded in 6-well plates, cultured until confluence in complete media without hygromycin B, and then incubated in serum-free DMEM for 24 h. Then, cell membranes were labelled by using EZ-Link Sulfo-NHS-LC-Biotin (Thermo Scientific, Waltham, MA, USA). After washing cells three times with PBS and 100 mM glycine to quench and remove excess biotin reagent, membrane proteins were isolated using a streptavidin pull-down (Pierce^TM^ High Capacity Streptavidin Agarose, Thermo Fisher Scientific, Waltham, MA, USA). Proteins were loaded and separated on a 10% Tris/glycine SDS gel. Afterwards, qualitatively equal gel slices were cut out from the gel. Proteins in the gel slices underwent tryptic digestion as previously described [58]. Generated peptides were applied to LC-MS/MS analysis. To achieve high sensitivity, a nano-LC-MS/MS setup was used that included a nano-LC system (EASY-nLC 1000, Proxeon—part of Thermo Scientific, Waltham, MA, USA) with an in-house packed C18 column (30 cm × 75 μm ID, ReproSil-Pur 120 C18-AQ, 1.9 μm, Dr. Maisch GmbH, Ammerbuch-Entringen, Germany) coupled online via a nano-spray flex ion source equipped with a PRSO-V1 column oven (Sonation, Biberach, Germany) to a Q-Exactive mass spectrometer (Thermo Scientific, Waltham, MA, USA). Peptides were separated with a reverse-phase chromatography using a 180 min binary gradient of water (A) and acetonitrile (B) containing 0.1% formic acid at 50 °C column temperature.

### 4.3. Proteomic Data Analysis 

The data were analyzed using Maxquant software (maxquant.org, Max-Planck Institute Munich, version 1.5.2.6 [59]) and searched against a reviewed canonical FASTA database of homo sapiens from UniProt (downloaded on December 12th 2014; comprising 16685 IDs), as previously described [21]. Trypsin was defined as protease and two missed cleavages were allowed for the database search. The option first search was used to recalibrate the peptide masses within a window of 20 ppm. For the main search peptide and peptide fragment mass tolerances were set to 4.5 and 20 ppm, respectively. Carbamidomethylation of cysteine was defined as a static modification. Protein acetylation at the N-terminus and oxidation of methionine were set as variable modifications. Only unique peptides were used for label-free quantification (LFQ). The Perseus software platform (http://www.perseus-framework.org, (accessed on 15 October 2020) copyright of Max Planck Institute of Biochemistry- Martinsried- Munich; Germany) was used to analyze the statistical significance of changes in protein levels between TIMP-3/HEK and control cells [60]. LFQ values were log_2_ transformed and a two-sided Student’s *t*-test was used for statistical analysis. A false discovery rate to 0.05 and s0 to 0.1 were set as the threshold for statistically significant alterations.

### 4.4. Methods to Validate Surfaceomics 

TIMP-3/HEK cells were maintained in complete media supplemented with 800 μg/mL hygromycin B. Then, TIMP-3/HEKs (or control HEK 293 cells) were seeded and grown in 6-well plates until confluence in complete media without hygromycin B. Finally, cells were incubated in serum-free medium for 24 h before levels of specific proteins were analyzed by Western blotting or FACS. Similarly, HEK 293 cells were grown to confluence and then incubated in serum-free medium containing 10 μM marimastat or DMSO for 24 h, before Western blotting analysis.

- Western blotting. Conditioned media were collected and proteins precipitated with 5% *v*/*v* trichloroacetic acid (Sigma, Aldrich, St. Louis, MO, USA) before being resuspended in Laemmli sample buffer (Bio-Rad, Hercules, CA, USA). Cells were collected with STET lysis buffer (50 mM Tris, pH 7,5, 150 mM NaCl, 2 mM EDTA, 1% Triton), containing protease inhibitor cocktail (1:500, P-2714, Sigma, Aldrich, St. Louis, MO, USA). Alternatively, cell membranes were labelled by using EZ-Link Sulfo-NHS-LC-Biotin and membrane proteins isolated, as described above (Section 4.2). Either conditioned media, lysate or membrane proteins were loaded onto an acrylamide gel and analyzed using SDS-PAGE electrophoresis, followed by immunoblotting. The following antibodies were used: anti-TIMP-3 (AB6000, Sigma, Aldrich, St. Louis, MO, USA), anti-EphA4 (targeting C-terminal EphA4, 6H7 Sigma, Aldrich, St. Louis, MO, USA; targeting the N-terminal EphA4, 35/EphA4, BD Biosciences, Franklin Lakes, NJ, USA), anti-calnexin (ADI-SPA-860-F, ENZO lifescience, Farmingdale, NY, USA), anti-GAPDH (88845, Cell Signaling, Danvers, MA, USA), anti-PTK7 (AF4499, R&D systems, Minneapolis, MN, USA), anti-APP (clone 22c11, Sigma, Aldrich, St. Louis, MO, USA), CADM1 (TSLC1 H-300, Santa Cruz Biotechnology, Santa Cruz, CA, USA), ADAM15 (HPA011633, Atlas Antibodies, Bromma, Sweden), SDC4 (ab24511, Abcam, Cambridge, UK), and actin (81178, Santa Cruz, Santa Cruz, CA, USA). For each experiment, 3 to 9 biological replicates were analyzed (number and raw quantifications are shown in Supplemental Materials). Bands corresponding to each protein were quantified using Image Lab software (Bio-Rad, Hercules, CA, USA) and normalized to the mean of the original non-normalized control values (HEK 293 cells for TIMP-3/HEK or DMSO-treated HEK 293 cells for marimastat-treated cells). A two-sided Student’s *t*-test was used to evaluate proteins statistically significantly regulated. A *p*-value less than 0.05 was set as the significance threshold.
- Cells were collected with ice-cold staining buffer (PBS 1X, FBS 2%, 2 mM EDTA), washed, and resuspended in 100 μL of staining buffer and incubated with appropriate antibodies for 30 min at 4 °C—LRP-1 (CD91-PE, Clone REA709, 130-111-412, Miltenyi Biotec, Bergisch Gladbach, Germany). After washing in PBS, cells were analyzed with a FACSCanto II flow cytometer (BD Biosciences, Franklin Lakes, NJ, USA).

### 4.5. Analysis of EphA4 mRNA Levels by qPCR

Total RNA was extracted from TIMP-3/HEK or HEK cells by using Qiagen RNeasy mini kit (Qiagen, Hilden, Germany) and reversely transcribed (1 μg) with Quantitect Reverse Transcription kit (Qiagen), following the manufacturer’s instructions. RT-qPCR was performed by using corresponding primers and SYBR Select Master Mix (Thermo Scientific) on a 7500 fast Real-Time PCR Detection System (Applied Biosystem, Part of Thermo Fisher Scientific, Waltham, MA, USA) according to the manufacturer’s instructions. The forward and reverse primers used for target genes were purchased from Bio-Rad (Unique Assay ID: qHsaCID0017961; Bio-Rad, Hercules, CA, USA). Relative fold change was calculated by first normalization with the reference gene *GAPDH* (Unique Assay ID: qHsaCED0038674, Bio-Rad, Hercules, CA, USA) and then against the level in control cells and presented as 2^−ΔΔCT^.

### 4.6. Exogenous TIMP-3 Induces Accumulation of EphA4 

TIMP-3/HEK or HEK controls were grown until confluence in 10 cm dishes prior to being incubated in serum-free medium for 48 h. Conditioned media were harvested and accumulation of FLAG-tagged TIMP-3 was confirmed using Western blotting (anti-FLAG M2; Sigma, Aldrich, St. Louis, MO, USA). HEK 293 cells were incubated for 24 h with conditioned media containing or not containing TIMP-3, then cells were collected in STET lysis buffer and levels of EphA4 analyzed using Western blotting.

### 4.7. Dose-Dependent Effects of Hygromycin B on EphA4 Levels

TIMP-3/HEK cells were maintained for 2 weeks in different concentrations of hygromycin B (0–800 μg/mL). Then, TIMP-3/HEKs were grown until confluence in 6-well plates, incubated for 24 h in serum-free medium, and harvested in STET lysis buffer. Levels of EphA4 in TIMP-3/HEKs were analyzed using Western blotting and compared to those in HEK controls cells. Similarly, HEK 293 cells were transfected with the empty pCEP4 expression vector and hygromycin B resistant cells were selected by adding 400 μg/mL hygromycin B to the growth medium. Hygromycin-resistant HEK 293 cells were seeded in 6-well plates, grown to confluence with or without 400 μg/mL hygromycin B, and then lysed with a STET buffer. Levels of EphA4 were evaluated using Western blotting. Viability of cells grown with different concentrations of hygromycin B was evaluated by CellTiter-Glo^®^ Luminescent Cell Viability Assay (Promega, Madison, WI, USA), following the manufacturer’s instructions. 

## Figures and Tables

**Table 1:** Type 1 transmembrane proteins significantly increased at the cell surface of TIMP-3 overexpressing cells.

Protein Name	Protein ID	Gene Name	*p*-Value	Ratio	MP Substrate
Desmocollin-1	Q08554	DSC1	1.86 × 10^−4^	8.40	unknown
Poliovirus receptor	P15151	PVR	8.86 × 10^−3^	5.56	unknown
Roundabout homolog 1	Q9Y6N7	ROBO1	3.11 × 10^−2^	5.13	[22]
Desmoglein-1	Q02413	DSG1	7.70 × 10^−4^	4.69	unknown
Protocadherin-7	O60245	PCDH7	1.13 × 10^−3^	4.29	unknown
Ephrin type-A receptor 2	P29317	EPHA2	1.87 × 10^−3^	4.01	[23]
Podocalyxin-like protein 2	Q9NZ53	PODXL2	3.19 × 10^−3^	3.87	[18]
Natural cytotoxicity triggering receptor 3 ligand 1	Q68D85	NCR3LG1	2.40 × 10^−2^	3.46	[24]
Receptor tyrosine-protein kinase erbB-2	P04626	ERBB2	5.72 × 10^−3^	2.98	[25]
Disintegrin and metalloproteinase domain-containing protein 15	Q13444	ADAM15	3.32 × 10^−2^	2.92	unknown
Trophoblast glycoprotein	Q13641	TPBG	2.18 × 10^−3^	2.87	unknown
Butyrophilin subfamily 2 member A1	Q7KYR7	BTN2A1	3.75 × 10^−2^	2.80	unknown
Inactive tyrosine-protein kinase 7	Q13308	PTK7	4.54 × 10^−3^	2.73	[26]
Plexin-B2	O15031	PLXNB2	1.78 × 10^−4^	2.73	[18]
Kin of IRRE-like protein 1	Q96J84	KIRREL	8.80 × 10^−4^	2.69	unknown
Desmoglein-2	Q14126	DSG2	2.53 × 10^−3^	2.67	[19]
Plexin-A1	Q9UIW2	PLXNA1	1.82 × 10^−4^	2.65	unknown
Nectin-2	Q92692	PVRL2	1.91 × 10^−2^	2.56	unknown
Prostaglandin F2 receptor negative regulator	Q9P2B2	PTGFRN	2.90 × 10^−5^	2.48	unknown
CD276 antigen	Q5ZPR3	CD276	9.90 × 10^−4^	2.11	[27]
Epidermal growth factor receptor	P00533	EGFR	1.43 × 10^−2^	2.03	unknown
Nicastrin	Q92542	NCSTN	6.48 × 10^−4^	2.02	unknown
Chondroitin sulfate proteoglycan 4	Q6UVK1	CSPG4	5.52 × 10^−2^	1.94	[28]
Receptor-type tyrosine-protein phosphatase F	P10586	PTPRF	2.45 × 10^−2^	1.91	[18]
Insulin-like growth factor 1 receptor	P08069	IGF1R	1.92 × 10^−2^	1.76	[29]
Basigin	P35613	BSG	1.79 × 10^−2^	1.76	[30]
Protocadherin-9	Q9HC56	PCDH9	4.60 × 10^−2^	1.66	[18]
Integrin beta-1	P05556	ITGB1	2.85 × 10^−2^	1.61	[31]

Protein name: proteins increased in TIMP-3/HEK above the FDR curve (FDR *p* = 0.05; s_0_ = 0.1) ID: UniProt accession number of the protein. Ratio: mean ratio of label-free quantification intensities between TIMP-3/HEK and control HEK293 cells (*n* = 3). *p*-value: for three biological replicates. MP substrate: proteins that undergo metalloproteinase-dependent shedding and their relative reference articles.

## Data Availability

The data presented in this study are available in Supplemental Materials.

## Supplementary Materials

Supplementary materials can be found at https://www.mdpi.com/1422-0067/22/5/2392/s1.

## Abbreviations

TIMPs	Tissue inhibitor of metalloproteases
ADAMs	A disintegrin and metalloproteinases
MT-MMP	Membrane-type metalloproteases
TNF	Tumor necrosis factor α
HEK	Human embryonic kidney
LC-MS/MS	Liquid chromatography-tandem mass spectrometry
EphA4	Ephrin type-A receptor 4
LRP-1	Low-density lipoprotein receptor-related protein 1
